# DNA Repair Deficiency in Breast Cancer: Opportunities for Immunotherapy

**DOI:** 10.1155/2019/4325105

**Published:** 2019-06-19

**Authors:** Elaine Gilmore, Nuala McCabe, Richard D. Kennedy, Eileen E. Parkes

**Affiliations:** ^1^Queen's University Belfast, Centre for Cancer Research and Cell Biology, 97 Lisburn Road, Belfast BT9 7AE, UK; ^2^Almac Diagnostics, 20 Seagoe Road, Craigavon BT63 5QD, UK

## Abstract

Historically the development of anticancer treatments has been focused on their effect on tumor cells alone. However, newer treatments have shifted attention to targets on immune cells, resulting in dramatic responses. The effect of DNA repair deficiency on the microenvironment remains an area of key interest. Moreover, established therapies such as DNA damaging treatments such as chemotherapy and PARP inhibitors further modify the tumor microenvironment. Here we describe DNA repair pathways in breast cancer and activation of innate immune pathways in DNA repair deficiency, in particular, the STING (STimulator of INterferon Genes) pathway. Breast tumors with DNA repair deficiency are associated with upregulation of immune checkpoints including PD-L1 (Programmed Death Ligand-1) and may represent a target population for single agent or combination immunotherapy treatment.

## 1. Introduction

Each individual cell endures hundreds of thousands of insults to its DNA each day [1]. Genomic instability is a pervasive feature associated with tumor cells and is the result of an accumulation of DNA damage within a cell [2]. Damage to DNA is triggered by many factors such as the generation of reactive oxidative species during metabolism (endogenous damage) and exposure to harmful environmental stimuli such as cigarette smoke or chemotherapy (exogenous damage) [3]. Efficient DNA damage responses such as cell cycle arrest and repair are therefore essential in order to maintain genomic integrity and stability [2].

DNA repair deficiency, in particular defects affecting the homologous recombination and Fanconi Anemia/BRCA repair pathway, is estimated to occur in 25% of breast cancers [4]. Notably, an estimated 60–69% of triple negative breast cancers (with absence of oestrogen receptor (ER) progesterone receptor (PR) as well as nonamplified HER2) are reported to have a defect in DNA repair, with features in common with BRCA1/2 mutated tumors described as “BRCAness” [5, 6].

Although loss of DNA repair pathways can result in tumor development, they can be exploited using targeted therapies. Moreover, the interaction of DNA damage with immune system activation and evasion provides novel therapeutic opportunities.

The roles of the host immune system and tumor microenvironment are now recognised as being crucial to the response to anticancer therapy [7]. The presence of infiltrating lymphocytes has been associated with improved outcomes in breast, ovarian, lung, colorectal and oropharyngeal cancers, and melanoma [8–11]. Notably triple negative breast cancer (TNBC) has been correlated with higher levels of lymphocytic infiltration compared to other subtypes of breast cancer [12]. Expression of the immune checkpoint Programmed cell Death Ligand-1 (PD-L1) is also increased in TNBC compared to non-TNBC [13].

The IMpassion130 study of the PD-L1 targeting antibody atezolizumab in combination with nab-paclitaxel demonstrated a significant improvement in overall survival in PD-L1 positive TNBC (22.0 vs 15.5 months) indicating the potential clinical impact of exploiting immunotherapies in this subgroup of breast cancer [14]. However, responses to immunotherapy are not restricted to TNBC, with responses observed in the neoadjuvant setting in both TNBC and hormone-receptor positive breast cancer [15], and in PD-L1 positive trastuzumab-resistant HER2 positive breast cancer [16].

A deeper understanding of the interconnectivity between DNA repair deficiency and immune response will enable rational trial design of single agent and combination immune checkpoint targeting therapies. Here we discuss how tumor cell intrinsic immune responses to loss of DNA repair result in modification of the tumor microenvironment and are associated with lymphocytic infiltration. In addition, chronic stimulation of immune pathways as a result of DNA repair deficiency favours an immunosuppressive microenvironment, with immune checkpoint upregulation, and may predict response to immune checkpoint blockade.

## 2. DNA Damage Repair Pathways

A series of interconnecting pathways exist within cells which function to repair DNA damage [17]. Although the DNA damage response is composed of different repair mechanisms which target distinct types of damage, they all encompass similar coordinated processes to detect DNA damage, recruit repair factors at the site, and then physically repair the damaged DNA [17].

In cancer cells, DNA repair mechanisms can be dysfunctional which leaves cells dependent on remaining pathways and therefore particularly vulnerable to therapies which target these specific pathways (Table 1) [18].

### 2.1. Base Excision Repair

Subtle changes to DNA such as single-strand breaks (SSBs) are repaired via the base excision repair (BER) mechanism [19]. This method of repair involves the removal of damaged bases form the double helix and the excision of the damaged section from the DNA structure [19]. Single nucleotide polymorphisms (SNPs) in members of the base excision repair pathway, XRCC1 and APE1, have been reported as contributing to increased risk of breast cancer, although population studies have not yielded consistent results [20, 21].

### 2.2. Nucleotide Excision Repair

Nucleotide excision repair (NER) is the mechanism responsible for the repair of single-strand lesions which cause a structural distortion within the DNA double helix [22]. Nucleotides surrounding the damaged site are excised and replaced by DNA replication machinery [17]. Defects in NER have been identified in early stage breast cancer and also reported to contribute to increased breast cancer risk women with exposure to cigarette smoke [23, 24].

### 2.3. Mismatch Repair

During replication, base mismatches can occur which distort the helical DNA structure [25]. These distortions are recognised by DNA damage response machinery which initiates the excision of the mismatched DNA, and the damaged site is then replaced with newly synthesised DNA [25]. Defects in mismatch repair (MMR) machinery are rarely seen in breast cancer, affecting 0.8–1.7% of women with breast cancer [26, 27] whereas MMR defects are seen in 15% of sporadic colorectal cancers [28]. There is now a known association between mismatch repair mutation and microsatellite instability with response to immune checkpoint therapies such as anti-PD-1; therefore identifying these women may be of clinical importance [29].

### 2.4. Nonhomologous End Joining

The repair mechanism nonhomologous end joining (NHEJ) is a simpler pathway which functions throughout the cell cycle to repair DSBs [30]. Repair is mediated by ligating the ends of the broken DNA strands together and therefore is prone to high rates of DNA deletion and mutation [17]. Two distinct NHEJ pathways are identified: classical and alternative NHEJ. Alternative NHEJ is a less-well-defined process which has been shown to have a higher probability of causing translocations and large deletions [31]. When faithful repair, via homologous recombination, is lost by mutation or epigenetic alterations to this pathway, repair of double-strand breaks is performed by NHEJ [32].

### 2.5. Homologous Recombination

Homologous recombination (HR) is one of the repair pathways responsible for the detection and repair of double-strand breaks (DSBs) [33, 34]. This mechanism of repair is often described as conservative as the original DNA sequence is restored at the damaged lesion [35]. The process of HR is largely restricted to the S and G2 phase of the cell cycle [36]. Nucleotides are excised both upstream and downstream of the damaged site and new DNA is synthesised using the homologous sister chromatid as a template [37]. HR defects occur in between 25 and 40% of breast cancers, from both germline and somatic mutations of key components of the HR pathway such as* BRCA1/BRCA2* [4, 6].

### 2.6. Fanconi Anemia/BRCA Pathway Loss

The Fanconi Anemia (FA)/BRCA pathway is a complex mechanism that involves the function of 19 genes and reestablishes DNA replication following DNA damage through the coordination of NER, translesional synthesis, and HR [38]. The FA/BRCA pathway is lost in approximately 25% of breast cancers due to mutation or silencing of one of constituent genes [4].

*BRCA1* was the first identified breast cancer susceptibility gene [39, 40] and is currently the newest member of the FA family. Biallelic mutations in* BRCA1* (typically embryonically lethal) were identified in a patient with early onset ovarian cancer with hypersensitivity to platinum based treatment and therefore deemed a new subtype of Fanconi Anemia (FANCS) [41].* BRCA2 *(*FANCD1*) was identified as a FA family member in 2002, following sequencing of* BRCA1* and* BRCA2* in cells from patients with FANCB and FANCD1 [42]. Mutations in other FA family members have been demonstrated to predispose to breast cancer, including* PALB2 (FANCN), BRIP1 (FANCJ), RAD51C (FANCO), SLX4 (FANCP), *and* FANCM* [43–50]. In summary, of the identified genes predisposing to hereditary breast cancer, the majority are FA family members.

### 2.7. Somatic Mutations of DNA Repair Genes in Breast Cancer

While* BRCA1* and* BRCA2 *are highly penetrant germline cancer predisposition genes, associated with familial breast cancers, somatic alterations also affect these genes [78–81]. Somatic mutations of the FA pathway also occur frequently in cancer and have been reported in 11.2% of breast cancers [82]. Promoter hypermethylation of* BRCA1 *has been reported in 13% of sporadic breast tumors [83], with promoter hypermethylation of* FANCC (PALB2), FANCO (RAD51C), *and* FANCF *also reported [84–86]. Collectively, somatic and germline mutations and alternations of BRCA and related HR genes result in a phenotype termed “BRCAness” [87]. However, there may be significant clinical variation in how germline vs somatic mutations and alterations behave in response to therapy, exemplified by improved response to carboplatin vs docetaxel observed in patients with germline* BRCA1* mutations but not in those with BRCA1 methylation or low mRNA expression [55]. However, while novel methods may allow variants of unknown significance and novel mutations of unknown pathogenic impact to be more clearly classified [88], taking this phenotypic approach to classification of* BRCA*-mutant-like HR-deficient cancers allows for clinical trial design targeting this subgroup of breast cancer.

### 2.8. Transcriptomic Identification of DNA Repair Deficiency

Tumors with loss of the FA/BRCA DNA repair pathway are sensitive to DNA damaging agents that cross-link DNA and stall DNA replication such as alkylating agents and anthracyclines. We previously identified a gene expression signature assay capable of prospectively identifying this distinct molecular subgroup of breast cancer patients with loss of the FA/BRCA pathway who benefited from chemotherapy [89]. Importantly, characterisation of the genes activated by loss of the FA/BRCA pathway revealed interferon-type immune gene signalling [90].

Consistent with this observation, both* BRCA1 *and* BRCA2* mutant breast cancers are known to be associated with lymphocytic infiltration [91, 92]. Cell line modelling demonstrates that loss of* BRCA1/2* results in upregulation of interferon related genes [93, 94]. Importantly the CXCL10/CXCR3 axis is activated in* BRCA*-mutant breast cancer and has been implicated in breast cancer progression and metastasis in both* in vivo *and clinical studies [95, 96].

## 3. Immune Response in Breast Cancer

A number of clinical trials have reported a favourable predictive and prognostic value of tumor infiltrating lymphocytes (TILs) in different pathological subtypes of breast cancer [9, 97, 98]. Lymphocytic infiltration is particularly recognised in tumors associated with genomic instability, such as those with a* BRCA1 *mutation [4, 91]. Increasing presence of TILs has been correlated with improved recurrence free survival following chemotherapeutic treatment of triple negative and HER2+ breast cancers [99]. In TNBC, a phase III clinical trial reported that each consecutive 10% increase in intratumoral and stromal TILs resulted in 15% reduced risk of recurrence and 17% reduced risk of cancer related death, irrespective of the type of chemotherapy administered [100]. However, in the same study increased TILs were predictive of poorer outcome in ER positive HER2 negative breast cancer. Notably, high FoxP3+ T-regulatory cells (T_regs_) have been associated with poorer outcomes in ER positive disease, yet improved outcomes in ER negative breast cancer [101, 102]. Examining lymphocytic infiltration as a whole may overlook the subtle effects of the different populations of lymphocytes present in the tumor and stroma.

Whereas BRCA1/2 mutant breast tumors have been recognised to be associated with increased lymphocytic infiltrate [87], early data suggests that loss of other DNA repair response proteins (for example, ATM) results in a markedly altered immune response and tumor microenvironment, with low levels of tumor infiltrating lymphocytes [103]. The evolution of the term “BRCAness” to describe a BRCA-mutant phenotype in tumors without BRCA1/2 mutations has enabled classification of this important subgroup of breast cancer but may overlook subtle differences in immune responses that may vary depending on specific “BRCAness” associated alterations. For example, although it is known that loss of heterozygosity may have a greater influence on tumor behavior than biallelic alterations resulting from two somatic events [88], the exact impact biallelic vs monoallelic alterations of HR-related genes may have on immune activation and response to immune blockade is unknown.

Despite the T-cell immune infiltration commonly present in* BRCA*-mutant and DNA damage response deficient breast cancers, tumor growth and invasion continue. Therefore DNA repair deficient tumors develop mechanisms of bypassing the antitumorigenic immune response, thriving in an inflamed microenvironment. The chronic inflammation mediated by DNA repair deficiency within the tumor microenvironment promotes cellular proliferation and invasion and, in addition, dysregulated pathways of immune equilibrium, thereby promoting immunosuppression [104–106].

### 3.1. STING Activation in DNA Damage Response Deficiency

Defects in DNA repair genes including* BRCA1 *and* ATM *have been shown to result in constitutive activation of the STimulator of INterferon Genes (STING) pathway in response to accumulation of cytosolic DNA [90, 107, 108]. Failed DNA repair results in the formation of micronuclei, within which cyclic GMP-AMP synthase (cGAS) colocalises with damaged DNA [109, 110]. Ruptured micronuclei result in activation of cGAS with subsequent synthesis of 2'3'-cGAMP which potently activates the STING pathway [111, 112]. Downstream activation of TANK-binding kinase 1 (TBK1) and interferon regulatory factor 3 (IRF3) then occurs, as well as canonical and noncanonical NF*κ*B pathways, resulting in upregulation of interferon stimulated genes [113, 114]. Interestingly, as well as activation of the STING pathway in DNA repair deficient cells, DNA damaging chemotherapies (including irinotecan, doxorubicin, and etoposide) and radiotherapy have similarly been demonstrated to activate the cGAS-STING immune response pathway [115–117].

STING agonists are now in early phase clinical trials in combination with immune checkpoint therapies based on their ability to induce immune responses in solid tumors [118, 119]. Activation of the cytosolic RNA-sensing RIG-I pathway has also been identified in breast cancer treated with doxorubicin [120], and similarly to STING agonists, RIG-I agonists are also in clinical development, with immunostimulatory effects on the tumor microenvironment and tumor clearance in murine models [121].

STING agonists cause upregulation of immune checkpoints including PD-L1 in the microenvironment [122], and upregulation of PD-L1 in response to DNA damage has been shown to be dependent on STING [90, 123]. PD-L1 expressing tumors (with PD-L1 identified on infiltrating immune cells ± epithelial cells) are more likely to respond to targeted immune therapies.

**Figure 1:**
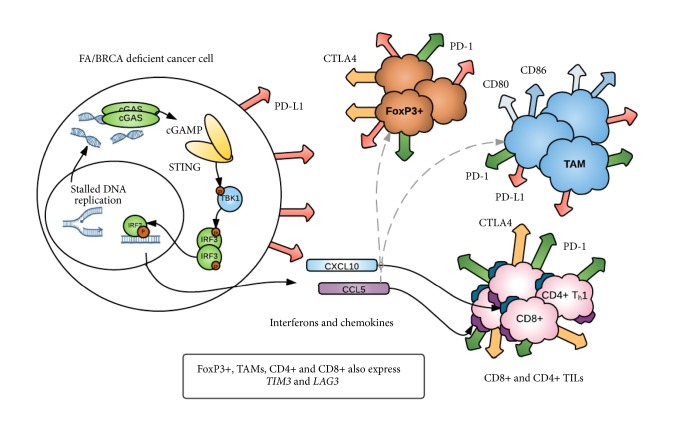
STING pathway activation in DNA repair deficient breast cancer. Stalled replication forks or damaged DNA as a result of mutations in Fanconi Anemia/BRCA repair pathway genes results in cytosolic DNA, detected by cGAS. 2'3'-cGAMP is produced, which then activates STING. STING dimerises or oligomerises, and TBK1 and IRF3 are phosphorylated. IRF3 then translocates to the nucleus resulting in the expression of immune genes including CXCL10 and CCL5. Note: other downstream activators of the STING pathway, notably TRAF6 and NF*κ*B, are not shown in this instance. CXCL10 and CCL5 are implicated in chemoattraction of CD8+ and CD4+ T-cells. However the tumor microenvironment may also contain immunosuppressive FoxP3+ CD4+ cells which express CTLA4, PD-1, PD-L1, LAG3, and TIM3; tumor-associated macrophages (TAMs) which express PD-1, PD-L1, CD80 and CD86, LAG3, and TIM3. Tumor infiltrating lymphocytes (TILs) may express CTLA4, PD-1, TIM3, and LAG3. Therefore, DNA repair deficiency results in activation of the cGAS-STING pathway which has both antitumorigenic and protumorigenic effects within the tumor microenvironment.

However, STING activation following radiotherapy has been shown to drive infiltration of immunosuppressive myeloid derived suppressor cells (MDSCs) [124]. In breast cancer, infiltration of MDSCs has been reported to promote progression and metastasis and may mediate resistance to immunotherapies [125]. Whether infiltration of these immunosuppressive cells is mediated by STING activation in breast cancer remains unclear. STING pathway activation may therefore have dichotomous effects on the tumor microenvironment. While STING activation in the acute phase is typically recognised to have an antitumorigenic immunogenic effect, chronic cGAS-STING activation may in fact result in an immunosuppressive microenvironment, activating the senescence associated secretory phenotype [126–128] and upregulation of immune checkpoints [90]. Moreover, chronic activation of cGAS-STING in chromosomally unstable tumors has been shown to result in STING-dependent metastasis [129]. The potential role of the STING pathway in the tumor immune microenvironment is illustrated in Figure 1.

### 3.2. Immune Checkpoints in Breast Cancer

Immune checkpoints are a number of inhibitory pathways within the immune system responsible for maintaining self-tolerance and modulation of the immune response [130]. Studies have reported that tumors are able to select particular immune checkpoint pathways to evade the immune system, particularly T-cells which target tumor antigens. This results in immune checkpoint proteins being frequently dysregulated in cancer [131].

When an antigen is recognised by the T-cell receptor, an immune response is initiated and then regulated by immune checkpoints via inhibitory and costimulatory signals [132]. Costimulatory receptor agonists or antagonists of inhibitory signals augment antigen-specific T-cell responses [133].

Although other forms of immunotherapy are also used in the clinical setting, the use of immune checkpoint targeted therapies has undoubtedly been remarkably successful, unleashing the potential of the antitumor immune response and revolutionising the management of human cancers [134]. Targeting the PD-1/L1 axis has been most fruitful in clinical trials, with many ongoing combination studies now using PD-1/L1 as a backbone of therapy (Table 2).

### 3.3. PD-1 and Ligands PD-L1/PD-L2

PD-1 is a transmembrane inhibitory coreceptor. Expression of PD-1 on T-cells and PD-L1 ligand interaction has been shown to have immunosuppressive functions in the tumor microenvironment [135]. PD-L2 expression is much more restricted than PD-L1 and so is mainly found on the surface of Antigen Presenting Cells (APCs) associated with its role in regulating the priming of T-cells [136].

PD-L1 expression is reported to be upregulated across a range of cancer types including breast, gastric, and lung cancers, although the significance of PD-L1 on prognosis and outcome remains uncertain in breast cancer [137, 138]. In the tumor microenvironment, PD-1/PD-L1 interaction results in T-cell death and inhibition of cytotoxic T-cell function [139]. Additionally, immunosuppressive Interleukin-10 (IL-10) production is stimulated [140]. Furthermore, PD-L1 expression enhances the conversion of helper T-cells (T_h_1) into immunosuppressive T_regs_ [141, 142]. Inhibiting the PD-1/PD-L1 pathway using PD-1 or PD-L1 targeting antibodies restores lymphocyte function and therefore cytotoxicity [143].

PD-L1 has been reported to be expressed epithelial cells in 20% of triple negative breast cancers [13] and has been proposed as a biomarker of response to immunotherapy. However the failure to respond in PD-L1 positive breast tumors (in up to 75% depending on the treatment setting) and the observed response in some PD-L1 low or negative tumors indicate that other markers of response need to be identified [134, 144]. The most promising of these in solid tumors has been the presence of microsatellite instability, leading to approval of immune checkpoint therapy in all advanced solid tumors with mismatch repair defects [145]. However, as discussed above, the incidence of these defects in breast cancer is low. Similarly tumor mutational burden (TMB) is a promising biomarker in other solid tumors, but most breast cancers do not typically demonstrate increased TMB [146].

Increased PD-L1 expression is identified in breast tumors deficient in DNA repair, and infiltrating immune-cell PD-1 and PD-L1 expression is higher in breast cancers with* BRCA1* or* BRCA2* mutations [90, 147]. Treatment with the DNA damaging agent doxorubicin results in increased expression of PD-L1 on breast cancer cells [148]. Interestingly, STING agonists given in combination with anti-PD-1 treatment result in improved responses in preclinical models [122].

Therefore, a close relationship is observed between DNA repair deficiency and upregulation of PD-L1 expression. Breast cancers with DNA repair deficiency, or BRCAness, may benefit from single agent immunotherapy targeting this pathway. However, independent of BRCAness, treatment of breast cancers with DNA damaging agents in combination with anti-PD-1/PD-L1 targeted therapy may result in enhanced tumor responses.

## 4. Immunotherapy in Breast Cancer

In metastatic TNBC, the combination of PD-L1 targeting atezolizumab with nab-paclitaxel resulted in a median 9.5-month improvement in overall survival (HR 0.62, 95% CI 0.45–0.86) in patients with PD-L1 positive immune infiltration [14]. In early stage breast cancer, neoadjuvant treatment of TNBC with anti-PD-1 in combination with chemotherapy resulted in an increase in pathological complete response (pCR) rates of 40% above expected [15]. These promising results indicate the potential of immunotherapy in breast cancer, although single agent anti-PD-1 treatment in the metastatic setting has not demonstrated a similar magnitude, with response rates of less than 20% in unselected advanced triple negative breast cancer, supporting combination approaches in future clinical trials [149].

Over 50 immune checkpoint therapy single agent and combination trials are ongoing in breast cancer, summarised in Table 2. The rate of translating these promising preclinical findings into the clinic is highly commendable and offers many patients a much-needed treatment option. However, the lack of an effective biomarker to select patients for immune checkpoint therapy exposes many patients who may derive no benefit from treatment to the risk of potentially serious immune mediated side effects, such as colitis, pneumonitis, liver toxicity, and durable endocrine effects including hypophysitis [150].

### 4.1. PARP Inhibitor and Immunotherapy Combinations in Breast Cancer

Poly(ADP-ribose) polymerase (PARP) inhibitors (inhibiting PARP1, involved in base excision repair) initially demonstrated efficacy in potentiating the effects of DNA damagers such as temozolomide [151]. Subsequently treatment with PARP inhibitors was found to result in synthetic lethality in BRCA1/2 mutant tumors [152, 153] and the PARP inhibitors olaparib and talazoparib are now FDA-approved as monotherapy treatments in BRCA1/2 mutant advanced breast cancer [154, 155].

As discussed above, the immune microenvironment of DNA repair deficient tumors is typically immunosuppressive with an exhausted T-cell infiltrate expressing high levels of checkpoints. However, as described by Yap and colleagues, the targeted cell death caused by PARP inhibitors has the potential to “reset” the tumor microenvironment and polarise the immune response towards a T_h1_ antitumorigenic profile, resulting in a shift from immune escape to elimination of the tumor [156]. Therefore PARP inhibitors represent a promising combination therapy with immune checkpoint targeting therapies.

PARP inhibitors have now been demonstrated in a number of preclinical studies to activate the innate immune cGAS-STING pathway [157–160]. These studies have further elucidated the mechanism of action of PARP inhibitors beyond synthetic lethality. Strikingly, treatment* in vivo* with the PARP inhibitor talazoparib in immunocompromised compared to immunocompetent models results in diminished responses [157]. Moreover, STING-dependent infiltration of CD8+ T-cells was demonstrated to be required for response to the PARP inhibitor olaparib [160]. These preclinical studies build a strong case for PARP inhibitor–immune checkpoint combination studies and the crucial role of the STING pathway in mediating immune responses. Interestingly these studies demonstrate a PARP inhibitor driven immune response in both HR-deficient and -proficient models [157, 160], supporting the rationale for PARP-immune checkpoint combinations beyond BRCA-mutant or HR-deficient disease.

In breast cancer, the combination of olaparib and durvalumab resulted in an overall response rate of 63% (95% CI 44–80%) at 28 weeks in 30 patients with germline BRCA1/2 mutations [161]. These promising results have led to the expansion of this study beyond germline BRCA-mutant disease to encompass homologous recombination deficient cancers [70]. In advanced TNBC the combination of niraparib and pembrolizumab demonstrated clinical benefit in 20 out of 46 patients, notably including 4 patients with no identified HR defect or detectable PD-L1 expression [162]. While it is likely that the dual combination of PARP inhibition and immune checkpoint blockade results in most marked responses in DNA repair deficient cancers, the addition of a third immune-stimulating or targeted agent may enhance responses in repair competent tumors. For example, the addition of antiangiogenic therapy may further stimulate an antitumorigenic immune response by inhibiting immunosuppressive effects of VEGF-A, which promotes infiltration of MDSCs and T_regs_ and prevents dendritic cell maturation [163]. A number of triplet combination studies, including PARPi, antiangiogenic and immune checkpoint blockade, are ongoing (Table 2).

## 5. Conclusions

It is clear that the immune system plays a significant role in tumor development, progression, and also response to therapy. Immune checkpoints are implicated in the process of immunosuppression and therefore represent ideal targets for therapeutic manipulation to encourage an antitumor immune response. As outlined here and elsewhere, there is a strong argument for the immune response to genomic instability as an independent biomarker in identifying candidates for immune targeting treatments [164].

DNA repair deficient breast cancer, identified using genomic or transcriptomic biomarkers of DNA repair, is associated with upregulation of immune checkpoints and an immune-cell infiltrated microenvironment. While activation of immune pathways such as STING in the acute phase promotes an antitumorigenic response, in the chronic phase DNA damage repair deficient tumors instead exploit this STING-mediated immune response, tailoring this to promote a proinvasive microenvironment favouring tumor growth. Moreover, this immune microenvironment can be further hijacked by chronic stimulation of pathways such as the senescence associated secretory phenotype, again favouring immunosuppression and immune escape [165].

As the immune microenvironment of chronically inflamed DNA repair deficient cancer consists of both antitumorigenic and immunosuppressive cell populations, therapies which therefore enhance the antitumor immune infiltration and activation, in combination with immune checkpoint therapies, represent a promising treatment strategy.

## Figures and Tables

**Table 1:** DNA repair pathways mutated in breast cancer and potential therapeutic interventions.

*DNA Repair Pathway*	*Defective mutation in Breast Cancer*	*Therapeutic Intervention*
Homologous recombination	BRCA1, BRCA2, ATM, ATR, CHK1, CHK2, BARD1, RAD51D, NBS1, PALB2, FANCD2, CtIP, PALB2 [17, 51–54]	Platinum based chemotherapies [55], PARP inhibitors (immune checkpoint blockade)

Non-homologous end-joining	DNA-PK, KU70/80 [56]	DNAPK inhibitors, ionizing radiation

Mismatch repair	MLH1, MSH2, MSH6, PMS2 [57, 58]	Immune checkpoint blockade

Base excision repair, Nucleotide excision repair, Translesional synthesis	APE1, XRCC1, ERCC2 [59, 60]	APE1 inhibitors [61]

**Table 2:** Current and completed clinical trials of immune checkpoint inhibition in breast cancer.

*Immunotherapy*	*Subtype*	*Target*	*Combination*	*Study*	*Phase*
Pembrolizumab	TNBC ER+/HER2-	PD-1	Single agent	NCT02555657 KEYNOTE-119 [62]	3

Pembrolizumab	BRCA mutated	PD-1	Single Agent	NCT03025035	2

Pembrolizumab	TNBC ER+/HER2-	PD-1	Single agent	NCT02447003 KEYNOTE-086 [63]	2

Pembrolizumab	TNBC ER+/HER2-	PD-1	Single agent	NCT01848834 KEYNOTE-012 [64]	1B

Pembrolizumab	TNBC ER+/HER2-	PD-1	Single agent	NCT02054806 KEYNOTE-028 [65]	1

Pembrolizumab	ER/PR-	PD-1	Single Agent	NCT03197389	1

Pembrolizumab	TNBC and HR+HER2-	PD-1	Decitabine + Soc NACT	NCT02957968	2

Pembrolizumab	TNBC	PD-1	EDP1503	NCT03775850	2

Pembrolizumab	TNBC	PD-1	Imprime PGG	NCT02981303	2

Pembrolizumab	HR+HER2-	PD-1	Eribulin	NCT03222856 KELLY [66]	2

Pembrolizumab	TNBC	PD-1	Chemotherapy	NCT01042379 I-SPY 2 [64, 67]	2

Pembrolizumab	TNBC	PD-1	Galinpepimut-S	NCT03761914	2

Pembrolizumab	TNBC	PD-1	Nab-paclitaxel + Epirubicin + Cyclophosphamide	NCT03289819	2

Pembrolizumab	TNBC	PD-1	Chemotherapy	NCT02622074 KEYNOTE-173 [68]	1B

Pembrolizumab	ER+HER2- / TNBC	PD-1	Radiation Radiation	NCT03366844	1

Pembrolizumab	Metastatic BC	PD-1	High Intensity Ultrasound	NCT03237572	1

Pembrolizumab	All	PD-1	Stereotactic Ablative Radiosurgery	NCT02303366 BOSTON II	1

Pembrolizumab	TNBC	PD-1	PVX-410 vaccine	NCT03362060	1

PDR001	TNBC	PD-1	Canakinumab CJM112 Trametinib EGF816	NCT02900664	1B

PDR001	TNBC	PD-1	LCL161 Everolimus Panobinostat QBM076	NCT02890069	1

PDR001	TNBC	PD-1	NZV930 NZV930 + NIR178	NCT03549000	1

Durvalumab	TNBC	PD-L1	Single agent Taxane-anthracycline chemotherapy	NCT02685059 GeparNuevo [69]	2

Durvalumab +/- Tremelimumab	All	PD-L1 +/- CTLA-4	Poly ICLC	NCT02643303	2

Durvalumab	BRCA mutated HER2-	PD-L1	Olaparib +Bevacizumab	NCT02734004 MEDIOLA [70]	2

Durvalumab	TNBC	PD-L1	Paclitaxel and Carboplatin	NCT03616886 SYNERGY	2

Durvalumab	BRCA mutated HER2-	PD-L1	Olaparib	NCT02734004 MEDIOLA [70]	1

Durvalumab	TNBC	PD-L1	Paclitaxel, Carboplatin and Oleclumab	NCT03616886 SYNERGY	1

Durvalumab	TNBC	PD-L1	Cediranib Olaparib Cediranib + Olaparib	NCT02484404	1

Atezolizumab	TNBC	PD-L1	Single agent	NCT01375842 [71]	1

Atezolizumab	TNBC	PD-L1	Nab-paclitaxel	NCT02425891 IMpassion130 [14]	3

Atezolizumab	HER2+	PD-L1	Trastuzumab Emtansine	NCT02924883 KATE2 [72]	2

Atezolizumab	TNBC	PD-L1	Cabozantinib	NCT03170960	1B

Atezolizumab	TNBC	PD-L1	RO7198457	NCT03289962	1

Nivolumab	TNBC	PD-L1	Romidepsin + Cisplatin	NCT02393794	2

Nivolumab	TNBC	PD-L1	Capecitabine	NCT03487666 OXEL [73]	2

Nivolumab	Metastatic	PD-L1	Nab-paclitaxel	NCT02309177	1

Nivolumab	All	PD-L1	COM701	NCT03667716	1

Avelumab	TNBC	PD-L1	Additional	NCT02926196 A-Brave [74]	3

Avelumab	TNBC	PD-L1	Utomilumab	NCT02554812 JAVELIN [75]	2

Avelumab	All	PD-L1	Utomilumab +/- Radiation Utomilumab + PF-04518600 PF-04518600 +/- Radiation Utomilumab + PF-04518600 + Radiation Cisplatin + Radiation	NCT03217747	2

FAZ053	TNBC	PD-L1	Single Agent PDR001	NCT02936102	1

LY3300054	HR+HER2-	PD-L1	Single Agent Ramucirumab Abemaciclib Merestinib LY3321367	NCT02791334	1

Tremelimumab	TNBC	CTLA-4	Monotherapy	NCT02527434 [76]	2

MSB0011359C	ER+ and/or PR+, HER2-	PD-L1 and TGF-*β*	Radiation	NCT03524170 RACHEL 1	1

LAG525	TNBC	LAG3	Single agent PDR001 / Carboplatin or combination	NCT03499899	2

Toripalimab	TNBC	PD-1	Single Agent	NCT02838823	1

TT1-621	All	CD47	Single Agent +PD1/PDL1 inhibitor +Pegylated interferon- *α*2a +T-Vec +Radiation	NCT02890368	1

Ipilimumab + Nivolumab	HER2-	CTLA-4 PD-1	Bicalutamide	NCT03650894	2

Ipilimumab + Nivolumab	HER2-	CTLA-4 PD-1	__	NCT03789110 NIMBUS	2

Epacadostat + Pembrolizumab	All	IDO-1 PD-1	INCAGN01876 (anti-GITR)	NCT03277352	1/2

Ipilimumab + Nivolumab	All	PD-1 PD-L1	Entinostat	NCT02453620	1

Nivolumab + Pembrolizumab + Atezolizumab	HER2+	PD-L1 PD-1 PD-L1	FT500 (Natural Killer cell)	NCT03841110	1

Ipilimumab + Nivolumab	All	CTLA-4 + PD-L1	Cryoablation	NCT02833233 [77]	N/A
